# Identification and Molecular Characterization of Molluskin, a Histone-H2A-Derived Antimicrobial Peptide from Molluscs

**DOI:** 10.5402/2012/219656

**Published:** 2012-10-15

**Authors:** Naveen Sathyan, Rosamma Philip, E. R. Chaithanya, P. R. Anil Kumar

**Affiliations:** Department of Marine Biology, Microbiology and Biochemistry, School of Marine Sciences, Cochin University of Science and Technology, Fine Arts Avenue, Kerala, Kochi 682016, India

## Abstract

Antimicrobial peptides are humoral innate immune components of molluscs that provide protection against pathogenic microorganisms. Among these, histone-H2A-derived antimicrobial peptides are known to actively participate in host defense responses of molluscs. Present study deals with identification of putative antimicrobial sequences from the histone-H2A of back-water oyster *Crassostrea madrasensis*, rock oyster *Saccostrea cucullata*, grey clam *Meretrix casta*, fig shell *Ficus gracilis*, and ribbon bullia *Bullia vittata*. A 75 bp fragment encoding 25 amino acid residues was amplified from cDNA of these five bivalves and was named “Molluskin.” The 25 amino acid peptide exhibited high similarity to previously reported histone-H2A-derived AMPs from invertebrates indicating the presence of an antimicrobial sequence motif. Physicochemical properties of the peptides are in agreement with the characteristic features of antimicrobial peptides, indicating their potential role in innate immunity of molluscs.

## 1. Introduction

Invertebrates mainly rely on their innate immune defenses to battle a variety of invading microbial pathogens. Among these molecules the antimicrobial peptides (AMPs) play an important role in the humoral innate immune mechanism of invertebrates. AMPs have received increasing attention in recent years as their contribution to host defense mechanisms and their potential as new pharmaceutical substances is becoming increasingly appreciated. This is mainly because of the broad spectrum activity of AMPs and the rapid development of microbial resistance to conventional antibiotics [1]. AMPs derived from histone proteins form an important category of peptide antibiotics [2]. Histone-derived antimicrobial peptides with potent activity have been isolated and reported from various organisms [3–11]. In the case of marine invertebrates histone-H2A-derived AMPs have been reported from Pacific white shrimp *Litopenaeus vannamei* [8], scallop *Chlamys farreri* [9], and abalone *Haliotis discus discus* [11]. From fishes histone-derived antimicrobial peptides have been reported from catfish *Parasilurus asotus* [4], Atlantic salmon *Salmo salar* [5], Atlantic halibut *Hippoglossus hippoglossus* [6], rainbow trout *Oncorhynchus mykiss* [7], and round whip ray *Himantura pastinacoides* [12]. Present study was carried out to identify histone-derived antimicrobial peptides from molluscs, namely, back water oyster *Crassostrea madrasensis*, rock oyster *Saccostrea cucullata,* grey clam *Meretrix *casta, fig shell *Ficus gracilis,* and ribbon bullia *Bullia vittata*.

## 2. Materials and Methods

### 2.1. Haemolymph Collection

Live *C. madrasensis* and *M. casta* were collected from Vembanad estuary, Kochi (Kerala, India) and *S. cucullata*, *F. gracilis*, and *B. vittata* from the coastal waters of Kochi. Samples were transported to laboratory in live condition. Haemolymph was collected from byssus muscles of bivalves and foot region of gastropods using 1 mL syringe rinsed in precooled anticoagulant solution (RNase free 10% sodium citrate, pH 7).

### 2.2. Isolation of Total RNA and cDNA Synthesis

Total RNA was isolated from haemolymph using TRI reagent. Purity and quality of RNA were checked on 0.8% agarose gel. RNA was quantified by spectrophotometry at 260 and 280 nm. Only RNA with absorbance ratio (A260 : A280) equal to or greater than 1.8 was used for the analysis. First strand cDNA was generated in a 20 *μ*L reaction volume containing 5 *μ*g total RNA, 1x RT buffer, 2 mM dNTP, 2 mM oligo d(T20), 20 U of RNase inhibitor, and 100 U of MMLV Reverse Transcriptase. The reaction was conducted at 42°C for 1 h followed by an inactivation step at 85°C for 15 min. Gene-specific primers forward (5′-atgtctggacgaggaaagggagga-3′) and reverse (5′-tacttggcaggtttctgggtct-3′) were used to amplify a product of 945 bp constitutively expressed gene, the beta-actin as an internal control.

### 2.3. PCR Amplification

Amplification of histone-H2A-derived antimicrobial peptide sequence from cDNA of molluscs was done using forward primer (5′-gaattcatgtctggacgaggaaaggg-3′) and reverse primer (5′-gcggccgcatagtttcccttacggagcaga-3′). PCR amplification of 1 *μ*L of cDNA was performed in a 25 *μ*L reaction volume containing 1x standard taq buffer (10 mM Tris-HCl, 50 mM KCl, pH 8.3), 1.5 mM MgCl_2_, 200 mM dNTPs, 0.4 mM each primer and 1 U Taq DNA polymerase. The thermal profile used was an initial denaturation at 94°C for 2 minutes followed by 35 cycles of 94°C for 15 seconds, 60°C for 30 seconds and 68°C for 30 seconds and a final extension at 68°C for 10 minutes. PCR products were analyzed by electrophoresis in 1.5% agarose gel in TBE buffer, stained with SYBR safe and visualized under UV light. Amplicons obtained were sequenced using ABI Prism BigDye Terminator Cycle Sequencing Ready Reaction kit on an ABI Prism 377 DNA sequencer at SciGenom Sequencing Facility, India.

### 2.4. Sequence Analysis

The nucleotide sequence and deduced amino acid sequence were subjected to BLAST at the NCBI (http://www.ncbi.nlm.nih.gov/blast). Translation of the cDNA was performed using the Expert Protein Analysis System (http://au.expasy.org/). Multiple sequence alignment of the peptide with previously reported histone-derived AMPs from other animals was performed with ClustalW. Homology searches were performed using BLASTn and BLASTp at National Center for Biotechnology Information (http://www.ncbi.nlm.nih.gov/). Physicochemical parameters of the deduced peptide were calculated by the ProtParam tool (http://cn.expasy.org/tools/protparam.html). The pdb data was generated by SWISS-MODEL [13–15] and three-dimensional arrangement of the peptide was created using PyMOL. Phylogenetic tree was constructed based on nucleic acid sequences by the neighbour-joining (NJ) method using MEGA version 5.05.

## 3. Results

**Figure 1:**
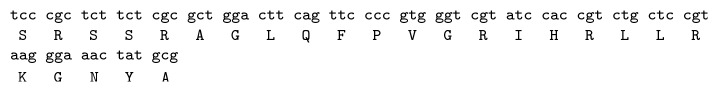
Nucleotide and amino acid sequences of histone-H2A-derived antimicrobial peptide, Molluskin from *C. madrasensis*, *S. cucullata*, *M. casta*, *F. gracilis*, and *B. vittata*.

**Figure 2:**
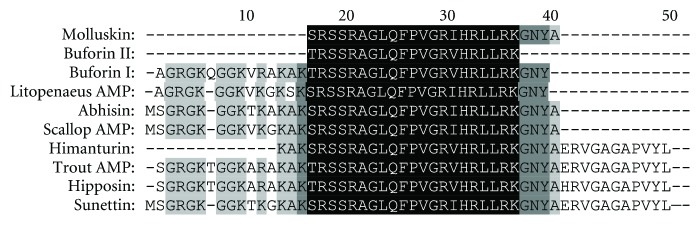
ClustalW multiple alignment of Molluskin (*C. madrasensis*, *S. cucullata, M. casta*, *F. gracilis*, and *B. vittata*) with Buforin I and II (*Bufo bufo gargarizans*), Hipposin (*Hippoglossus hippoglossus*), rainbow trout H2A (*Oncorhynchus mykiss*), Litopenaeus AMP (*Litopenaeus vannamei*), scallop AMP (*Chlamys farreri*), Abhisin (*Haliotis discus*), Sunettin (*Sunetta scripta*), and Himanturin (*Himantura pastinacoides*).

**Figure 3:**
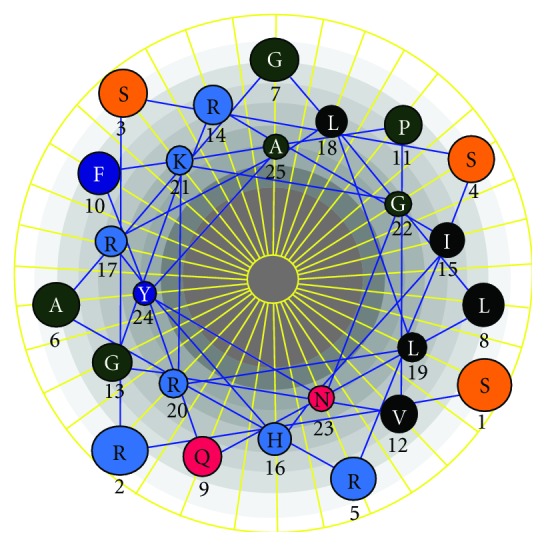
Schiffer-Edmundson helical wheel diagram demonstrating probable amphipathic *α*-helical conformation of Molluskin.

**Figure 4:**
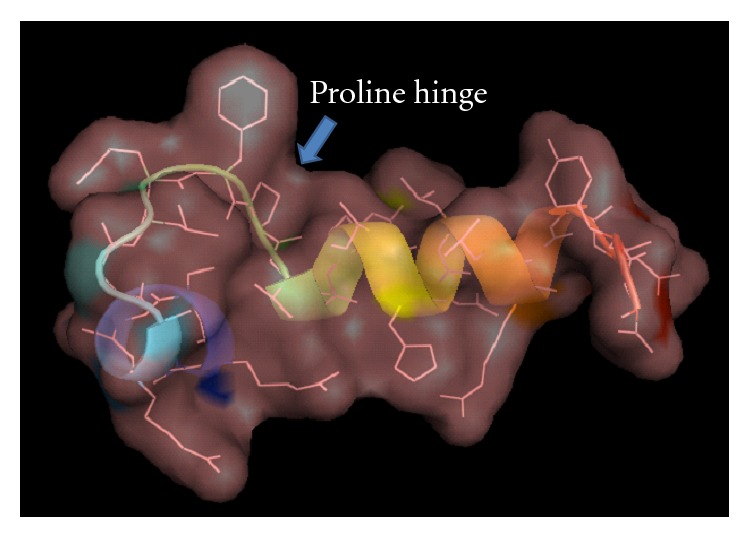
Predicted 3-dimentional structural arrangement of Molluskin using PyMol. Presence of proline hinge is highlighted.

**Figure 5:**
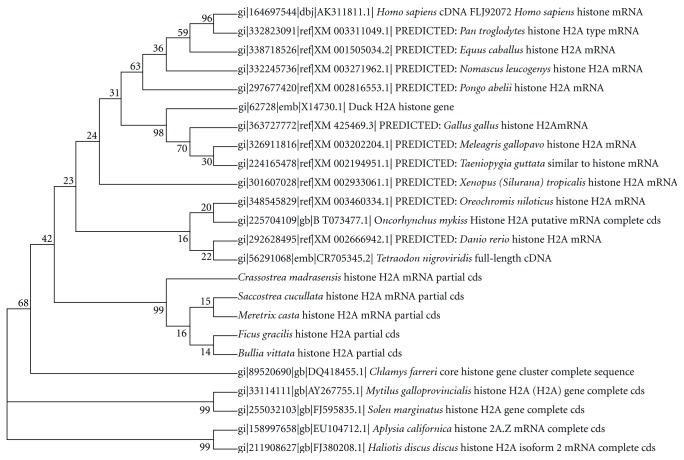
A bootstrapped neighbor-joining tree obtained using MEGA version 5.05 illustrating relationships between the nucleotide sequence of *Crassostrea madrasensis*, *Saccostrea cucullata*, *Meretrix casta*, *Ficus gracilis*, and *Bullia vittata* to the nucleotide sequences of previously reported histone-H2A from different organisms.

A 75 bp fragment cDNA encoding 25 amino acids from the mRNA of the five molluscs was obtained by RT-PCR (Figure 1). BLAST analysis of the nucleotide and deduced amino acid sequences revealed that the peptides belonged to histone-H2A family. The H2A sequence obtained was similar for all the five molluscs (2 oysters, 1 clam, and 2 gastropods). The obtained nucleotide and deduced amino acid sequences were deposited in GenBank database: *B. vittata* (GenBank ID: HQ720143), *C. madrasensis* (GenBank ID: HQ720145), *F. gracilis* (GenBank ID: HQ720146), *S. cucullata* (GenBank ID: HQ720147), and *M. casta* (GenBank ID: HQ720148). Multiple sequence alignment of the amino acid sequence with previously reported histone-H2A-derived AMPs revealed that the 25 amino acid sequence of the deduced peptide showed similarity to previously reported histone-H2A-derived AMPs like Buforin I, Buforin II, Hipposin, Himanturin, Abhisin, Sunettin, and those reported from *Litopenaeus vannamei* and *Chlamys farreri* (Figure 2). This H2A-derived peptide sequence amplified from *C. madrasensis*, *M. casta*, *S. cucullata*, *F. gracilis*, and *B. vittata* was termed as “Molluskin.” Sequence analysis of the peptide was carried out using ProtParam software which predicted Molluskin to have a molecular weight of 2.84 kDa and a theoretical isoelectric point (pI) of 12.18. ProtParam estimated the half life of peptide to be 1.9 hours in mammalian reticulocytes, more than 20 hours in yeast and more than 10 hours in *E. coli*. Molluskin was found to be rich in arginine (20%), leucine (12%), serine (12%), glycine (12%), and alanine (8%) as reported in all other histone-H2A-derived AMPs. The 25 amino acid peptide was found to have a net charge of +6. Hydrophobicity of Molluskin was found to be +21.92 kcal/mol (32%) as predicted by PepDraw. Analysis of Molluskin using Protean module of the DNASTAR Lasergene sequence analysis software suite revealed that the peptide will have a concentration of 1.91 mg/mL for an absorbance of 1 OD measured at 280 nm and 1 *μ*g of the peptide would contain 352.08 pmoles. Hydrophobic amino acids constituted 29.61% while polar amino acids represented 23.47% of the total weight of Molluskin. Schiffer-Edmundson helical wheel modeling of the peptide using Protean module revealed clustering of hydrophobic and hydrophilic/basic residues on opposing sides of the helical wheel (Figure 3). This result suggests an amphipathic nature and an *α*-helical structure for the Molluskin. Three-dimensional arrangement of the peptide generated in PyMOL is presented in Figure 4. Analysis of Molluskin for antimicrobial activity was carried out with antimicrobial peptide database (http://aps.unmc.edu/AP/main.php) which predicts Molluskin to be an antimicrobial peptide with a protein-binding potential of 2.96 kcal/mol. Bootstrap distance tree calculated confirmed the similarity of nucleotide sequences to the previously reported histone-H2A nucleotide sequences (Figure 5).

## 4. Discussion

In invertebrates, humoral immunity mainly consists of antimicrobial agents present in the circulating body fluid [16]. Therefore identifying novel antimicrobial peptides provides information crucial for elucidating invertebrate innate immunity. Molluskin exhibited significant similarity with previously reported histone-H2A-derived AMPs as indicated in Figure 2. Molluskin has a Ser at N-terminus region. H2A-derived antimicrobial peptides reported from other invertebrates and Himanturin reported from round whip ray also possess Ser at the corresponding position, but in case of all other vertebrates, Thr is present in position of Ser. Molluskin possesses Ile at position 15 from N-terminus. This is the same for all invertebrates as they possess Ile at the corresponding position, whereas in vertebrates Val occupies the position instead of Ile. Since Ser and Thr are hydrophilic and Ile and Val are hydrophobic and since they do not contribute to the charge of the peptide, their interchange will have no or very little effect on the activity of the peptides. All these antimicrobial peptides are derived from the N-terminal region of H2A histone suggesting its role in the innate immunity of an organism.

Histone-H2A-derived antimicrobial peptides are cleaved from their precursors mainly by the action of proteolytic enzymes. In Asian toad *Bufo bufo gargarizans*, the intact histone-H2A protein is secreted into the stomach and Buforin I is produced by the action of pepsin isozymes cleaving the Try 39-Ala 40 bond of intact protein [17]. Similarly in catfish (*Parasilurus asotus*), parasin I is produced by cleavage of Ser19-Arg20 bond of histone-H2A by cathepsin D found in skin mucus of the fish [18]. To understand the action of proteolytic enzymes on histone-H2A protein of molluscs, we considered a full length histone-H2A protein sequence of *Sunetta scripta* (GenBank ID: HQ720149) previously reported by us (in press: Sathyan et al. 2012; Identification of a histone-derived, putative antimicrobial peptide sunettin from marine clam *Sunetta scripta*. Blue Biotechnology Journal). The sequence was analyzed using PeptideCutter tool (http://web.expasy.org/peptide_cutter/). PeptideCutter tool predicts extracellular digestive enzyme, trypsin to have potential cleavage site at amino acid position 15 and enzymes proteinase K and Asp-N endopeptidase to have a potential cleavage site at amino position 40 from the N-terminus. Proteolytic activity of these enzymes will result in the formation of a peptide like the Molluskin. Enzyme pepsin was found to have a cleavage site at amino acid position 39 from the N-terminus, which would release a peptide similar to Buforin 1, Abhisin, and also to the histone-H2A-derived AMPs from* Litopenaeus vannamei* and *Chlamys farreri*. These findings suggest that the proteolytic enzymes could transform the N-terminus of histone-H2A in molluscs into an active antimicrobial peptide assisting in the innate immunity of the organisms. AMPs derived from precursors are less understood in case of marine invertebrates and therefore the study would provide a better understanding of their innate immune responses. Two distinct pathways, the Toll pathway and Immune Deficiency (IMD) pathway, mediate the secretion of antimicrobial peptides in *Drosophila*. Infections mainly due to fungal and Gram-positive bacterial attack activate the Toll pathway, whereas the IMD pathway is activated, predominantly, in response to infections by Gram-negative and other Gram-positive bacteria [19, 20]. A pathway similar to that of *Drosophila* IMD, termed as LvIMD, was reported from a marine invertebrate *Litopenaeus vannamei* [21]. Existence of Toll receptors was also reported in *Litopenaeus vannamei* [22, 23]. Mechanisms similar to these might be involved in the cleavage of precursor-derived antimicrobial peptides and detailed studies in this area would open up new frontiers in AMP research.

Broad spectrum activity against bacteria and fungi was exhibited by histone-H2A-derived antimicrobial peptides reported from various sources. Antimicrobial peptides are also viewed as agents with therapeutic potential against cancer cells [24]. Hipposin and Buforins are the most studied Histone-H2A-derived antimicrobial peptides. Hipposin exhibited strong antibacterial activity against several Gram-positive and Gram-negative bacteria and activity could be detected down to a concentrations of 1.6 *μ*g/mL [6]. Buforins are among one of the most potent antimicrobial peptides. In addition to their broad spectrum activity against bacteria and fungi [3], they also possess antiendotoxic and anticancer activities [25]. General mode of action of antimicrobial peptides is to kill cells through membrane disruption. Buforin II differs from this generalization as it does not cause significant membrane permeabilization [26]. Instead, Buforin II appears to readily enter bacterial cells *in vivo* [27] and lipid vesicles *in vitro* [26] showing that the peptide can traverse the cell membrane without any receptor. Once inside, it is believed to cause bacterial cell lysis by interacting with intracellular nucleic acids [28]. No cytotoxic activity against normal mammalian cells was observed for Buforin II [29]. NMR structural studies showed that proline at position 11 serves as a hinge between a C-terminal helix and N-terminal-extended helical structure [30]. This sole proline residue (Pro_11_) of Buforin II is necessary for effective translocation across cell membrane [26, 27]. Presence of a proline hinge as in Buforin II was also found to be a characteristic feature of Molluskin (Figure 4). We suppose that the presence of proline hinge clearly indicates that the antimicrobial activity of Molluskin lies in its ability to interact with nucleic acid rather than membrane permeabilization. Buforin II exhibits selective cytotoxicity against cancer cells through interaction with cell surface gangliosides and once inside the cell they induced mitochondria-dependent apoptosis [31]. Structural similarity of Molluskin to Buforin II may imply potential anticancer activity. Antimicrobial activity of Molluskin was further confirmed by antimicrobial peptide database (http://aps.unmc.edu/AP/main.php) which predicts it to be an AMP since it forms alpha helices and has at least 5 residues on the same hydrophobic surface which allows the peptide to interact with membranes. Molluskin shows the characteristic features of AMPs including high cationicity (+6), higher hydrophobic residue (32%), and 2.96 kcal/mol protein-binding potential. 

The molecular phylogenetic tree based on nucleic acid sequences of previously reported histone-H2A-derived AMPs demonstrates that the members of the family are derived from a common ancestor by a series of evolutionary changes (Figure 5). The boot strap distance tree calculated reveals that nucleotide sequences of Molluskin from all five molluscs align with the molluscan group. Histone genes evolve very slowly and therefore, evolutionary analyses of histones should be informative with regard to the phylogenetic relationships of distantly related organisms [32].

## 5. Conclusion

Peptide having antimicrobial sequence motif was identified from the histone-H2A of *C. madrasensis*, *S. cucullata*, *M. casta*, *F. gracilis*, and *B. vittata* and was named as Molluskin. High similarity of Molluskin in terms of physicochemical properties and molecular structure to other histone-H2A-derived AMPs with proven antimicrobial activity strongly endorse it to be an antimicrobial peptide. This work was undertaken to study the presence of histone-derived AMPs in molluscs depicting its possible role in innate immunity. Synthesizing histone-derived AMPs for commercial applications would be a highly promising endeavor as an alternative to the conventional antibiotics which elicit drug resistance in microbes and impose tremendous ecological damage to the environment both terrestrial and aquatic. Since Molluskin is a very short peptide, it has the potential to be developed into an effective antimicrobial agent for use in aquaculture and medicine.
